# A Critical Overview of Mental Health Policies for the Treatment of Schizophrenia in Chile

**DOI:** 10.62641/aep.v53i5.1952

**Published:** 2025-10-05

**Authors:** Diego Atencio-Quevedo, Alejandra Caqueo-Urízar, Juan-Carlos Romero-Romero

**Affiliations:** ^1^Escuela de Psicología y Filosofía, Universidad de Tarapacá, 1000000 Arica, Arica y Parinacota, Chile; ^2^Instituto de Alta Investigación, Universidad de Tarapacá, 1000000 Arica, Arica y Parinacota, Chile

**Keywords:** Schizophrenia, health policy, regional health strategies

## Abstract

**Background:**

The Universal Access to Explicit Health Guarantees (AUGE) program in Chile provides clinical guidelines for the treatment of the first episode of Schizophrenia. This study contextualizes these guidelines within Chilean mental health policies and the theoretical framework of cultural biology, examining the balance between biomedical models and person-centered approaches.

**Methods:**

Critical discourse analysis was employed to explore the AUGE guidelines, identifying strengths and limitations in their formulation and implementation. This methodology allowed for the examination of the underlying power dynamics and ideological assumptions embedded within the guidelines.

**Results:**

The findings reveal that the Clinical Guidelines reinforce a normative character through grammatical constructions that often present patients as objects of intervention. Analysis of the social context shows an emphasis on a biomedical perspective and pharmacological interventions, potentially overshadowing rights-based and gender approaches in favor of international guidelines which may not be adequate in Chilean context. The guidelines also lack specific considerations for cultural particularities of ethnic minority, indigenous, and rural individuals. Finally, the guideline’s narrative emphasizes deficits and dysfunctions, potentially contributing to stigma and positioning health professionals as the primary decision-makers. This may limit a truly participatory and empowering model of care.

**Conclusion:**

The study underscores the necessity of a shift towards relational trust and co-construction in therapeutic practices. Recommendations on the formulation of mental health care Guidelines for Schizophrenia are proposed. Future guidelines should focus on increasing funding and support for community-based mental health services, implementing mandatory training for healthcare professionals on person-centered and culturally sensitive approaches and establishing mechanisms for ongoing patient and community feedback to ensure policy responsiveness and relevance.

## Introduction

Schizophrenia is one of the leading causes of long-term disability worldwide, causing significant impairment in the well-being and quality of life of those diagnosed [1, 2]. Despite the existence of evidence-based treatments, a considerable number of people fail to participate satisfactorily in work or educational settings or to achieve economic and social independence [3].

Until the 1970s, the conceptualization of Schizophrenia was strongly linked to a notion of chronic, degenerative and incurable mental illness, characterized by a break with reality, hallucinations, delusions and cognitive impairment [4, 5], where the therapeutic approach focused on institutionalization of people with this diagnosis and treatments that generated severe side effects rather than addressing the underlying causes of the disorder [6].

This progressively changed as a result of advances in neurobiological and psychosocial research, recognizing the complexity of Schizophrenia and the importance of environmental and psychosocial factors in its development and course [7].

This paradigm shift led to the development of new, more effective and less invasive pharmacological and psychosocial treatments, recognizing the importance of empowering patients in their recovery process and emphasizing the need to provide psychosocial support, rehabilitation and opportunities for community participation. In this way, it is understood that recovery is a plausible goal and that people with Schizophrenia can lead full and meaningful lives [8].

### Recovery in Schizophrenia

In the context of Schizophrenia, improvement in recovery indicators traditionally involves achieving adequate social, occupational and personal functioning, where the promotion of optimism, access to employment and empowerment of patients in their recovery process play a central role [8].

Based on the above, only a small proportion of people with Schizophrenia achieve full recovery, motivated both by individual factors of the users, and largely due to gaps, inequities and sociocultural disparities that hinder access to treatment, posing major challenges in terms of achieving not only symptomatic remission, but also a degree of functioning and inclusion that allows a good quality of life [9].

### Schizophrenia and Public Policies in Chile

Among the main milestones in mental health in the country, it is worth mentioning the presentation of the first National Mental Health Policies and Plan in 1993 and the adoption of a community model of health care, considered as a response that ensured the human rights of patients in the face of the failure of the asylum model [10].

Between 2000 and 2017, significant progress was made in the implementation of this model, creating community devices, increasing mental health staffing and de-institutionalizing patients who lived in psychiatric hospitals [11].

In the case of Schizophrenia, it was included in the Universal Access to Explicit Health Guarantees (AUGE) in 2005, which meant a commitment from the State to guarantee access to a prompt and accurate diagnosis and treatment for people with this disorder. In this context, the first version of the AUGE Clinical Guidelines for the Treatment of People from the first episode of Schizophrenia was published in 2005 [12], becoming a fundamental tool to standardize care and ensure its effectiveness.

The development of this clinical guideline involved a multidisciplinary team of mental health experts and representatives of patients and their families. The purpose of forming this team was to integrate different perspectives and ensure that the guideline was relevant and applicable to the Chilean reality, recognizing the need for a comprehensive approach to improve the quality of life of people with Schizophrenia [13].

Currently, the third version of this guideline, published in 2017 [13], is in use. The previous editions, released in 2005 and 2009, laid the groundwork for managing the condition, and this new version seeks to refine and update the recommendations based on the most recent scientific and clinical advances. While many of the recommendations from the 2009 edition remain relevant, the 2017 edition focuses on specific areas in need of updating and incorporates new knowledge gained since the last publication, ensuring that treatment guidelines are aligned with contemporary best practices.

### The Present Study

In Chile, the lifetime prevalence of Schizophrenia is estimated to be 9 in 1000 people [14], with more than 28,000 people being treated under the AUGE Schizophrenia program [13], and an incidence of 18.9 per 100,000 person-years of non-affective psychotic disorders, which are treated as part of the First-Episode Schizophrenia Program [15]. Despite advances in mental health care in the country with the implementation of the community model, important challenges remain, such as insufficient resources, unequal access to services, and the need for greater participation of patients and their families in decisions about their treatment.

In this context, cultural biology, developed by Maturana and Dávila [16, 17], offers a valuable perspective for understanding Schizophrenia and its management. This theory proposes an integrated view of the human being, where biology and culture influence each other in a continuous process of co-evolution. Language plays a crucial role in this interaction, as it not only enables communication, but also shapes the way we think and relate to the world.

Cultural biology invites to understand how mental health problems, such as Schizophrenia, become political priorities, how decisions around them are made, and how public policies are implemented and evaluated [16]. It also allows to analyze the dominant narratives about the disorder, the underlying power relations that influence the health care system, and the role assigned to patients in their own recovery process.

The theoretical contributions of cultural biology conceptualize the human being as an autopoietic system (that produces and maintains itself) constituted through interaction, emotion and language [16, 17]. Knowledge, from this point of view, is not the reflection of an objective external reality, but a consensual coordination of actions and meanings that emerge in the relational realm [16, 17]. Therefore, mental health cannot be reduced to neurological or chemical dysfunctions but should be understood as a relational and emotional process that occurs in the context of one’s life history and social environment.

A central premise of this approach is the understanding of love as the biological foundation of human coexistence, defined as the emotion that allows the recognition of the other as a legitimate being in the relationship [16]. Therapeutic transformation, then, implies the recovery of legitimacy and trust. Based on this, the actions that mental health care services should provide include the incorporation of affective accompaniment, spaces of shared meaning, the inclusion of people with the lived experience of Schizophrenia in the design and evaluation of public policies, and the training of professionals in these epistemological principles.

Incorporating the perspective of cultural biology in the analysis of public policies and regulations in mental health opens new possibilities and makes it possible to raise questions about traditional practices, identify power dynamics, and promote an approach that respects the subjectivity and agency of people living with Schizophrenia.

Thus, this article proposes to critically analyze public policies and technical regulations for the diagnosis and treatment of Schizophrenia, with the aim of identifying (a) the notions made in them about the recovery process; (b) the space given to the subjectivity (the individual’s unique experience, perspective, feelings and thoughts) and agency (the capacity of individuals to act independently and to make their own free choices) of the patient in their treatment; (c) the power dynamics between the State and the actors of the mental health care system and; (d) points of improvement of the AUGE program.

## Methods

This study is based on a qualitative approach to Critical Discourse Analysis, according to Norman Fairclough’s postulates, which explores how language is not simply a neutral means of communication, but a powerful tool that shapes, reproduces, and challenges power relations and ideologies in society [18]. For these purposes, the analysis is carried out using a three-dimensional model that examines any discursive practice on three interrelated levels: text, discursive practice, and social practice.

The first corresponds to the description of the linguistic characteristics of the discourse, such as the vocabulary used, grammatical structure of the sentences, cohesion mechanisms that connect the different parts of the text and the way in which the discourse is organized.

The second level involves an interpretation of the social context in which the discourse is produced, analyzing who the participants are, what roles they play and how they relate to each other. It also examines the relations between the text and the institutional context, the social structures, ideologies and values reflected in the text under analysis.

Finally, the third stage corresponds to explanation, where discourse analysis is related to social and power structures. In this stage, the aim is to understand how discourse contributes to reproducing or transforming existing power relations, how it constructs or challenges social identities, and how it influences the way people think and act.

The analyzed document corresponds to “Guías Clínicas AUGE. Tratamiento de personas desde el primer episodio de Schizophrenia”, published by the Department of Mental Health of the Division of Disease Prevention and Control, belonging to the Undersecretariat of Public Health of the Ministry of Health of Chile. Table 1 shows the distribution of the document.

**Table 1.** **Composition of “Universal Access to Explicit Health Guarantees (AUGE) Clinical Guidelines for the Treatment of Persons with First Episode of Schizophrenia” (Chilean Ministry of Health; June, 2017)**.

Chapter	No. of pages	No. of sections	No. of subsections
1. Clinical decision algorithm	1	NA	NA
2. General recommendations	3	1	NA
3. Introduction	21	8	NA
4. Scope of the guidelines	3	3	NA
5. Objectives	1	NA	NA
6. Methodology	2	NA	NA
7. Clinical questions addressed in the guide	1	3	NA
8. Clinical aspect: screening and diagnostic confrontation	14	8	NA
9. Clinical aspect: treatment in acute and maintenance phase of schizophrenia	12	2	10
10. Clinical aspect: treatment of resistant schizophrenia	4	5	NA
11. Clinical aspect: psychological and psychosocial treatment of schizophrenia	5	10	NA
12. Clinical aspect: treatment considerations in pregnant and breastfeeding women	3	3	NA
13. Dissemination	1	NA	NA
14. Guideline development	3	3	NA
15. Development of clinical questions and key messages	17	NA	NA
Annexes	13	5	NA
Glossary	1	NA	NA
Total	105	51	10

NA, not applicable.

## Results and Discussion

### Analysis of the Discourse Structures

It is observed that in the Clinical Guidelines a normative character of the document is reinforced. Linguistic choices such as *should* and *must*, reinforce a normative authority that guides clinical decisions. However, it is important to note that the guideline recognizes the need to adapt the recommendations to the specific context of each patient and the importance of reaching consensus with the user, which introduces flexibility in its application.

From the perspective of cultural biology, this flexibility can be interpreted as an openness to the coordination of consensus in relational living, a key principle in the construction of human systems that seek to transcend rigid structures to promote the responsible autonomy of those involved [16, 17, 19]. Recent studies in clinical decision-making demonstrate how cultural background influences preferences for shared decision-making and patient autonomy [20], and how the role of the physician has evolved as a consultant, emphasizing the importance of recognizing the patient as an active agent in their own care [21].

Additionally, grammatical constructions are observed that present patients as objects of intervention (“the user should continue to be controlled […]”), which reinforces a passive position of the user in the treatment process, subtracting agency and participation in decision making.

From cultural biology, this discursive construction contrasts with the reflective autonomy proposal, where people are seen as active subjects in their life history. A person-centered praxis should incorporate language that encourages the co-design of care experiences, emphasizing the agency and decision-making power of patients in their recovery [16, 17, 19].

Finally, in terms of intertextuality, the guideline cites a variety of documents, including the International Statistical Classification of Diseases and Related Health Problems (ICD-10) [22] and National Institute for Health and Care Excellence guidelines [23]. While this endorsement in scientific literature reinforces the legitimacy of the guideline, it also suggests a reliance on international perspectives that may not be fully applicable to the local context.

In this regard, it highlights the importance of the cultural identity and relational history of individuals and communities as the basis for any intervention [20]. Systematic reviews have been conducted that integrate clinical practice guidelines with methodological studies to update and strengthen the validity and clinical applicability of recommendations [24], in that way the adoption of international standards can be aligned with local realities through a process of active listening and mutual respect, a fundamental principle in building an environment of trust and collaboration [16, 17, 24].

### On the Social Context

The analysis of the social context of the Clinical Guidelines reveals a construction deeply influenced by the Chilean institutional and healthcare environment. Developed by the Ministry of Health under the framework of the GES program, its central purpose is to optimize the diagnosis and treatment of Schizophrenia, promoting rights-based healthcare and efficient use of health resources. However, the approach adopted reflects a dominant biomedical perspective that prioritizes pharmacological and technical interventions, a framework that is inserted in a sociocultural context where mental illness still faces stigma and where inequities in access to mental health are significant.

Furthermore, although the importance of the rights and gender approach is mentioned, these aspects seem to play a secondary role compared to biomedical considerations, which evidences the tensions between an ideal of person-centered care and a clinical practice still influenced by traditional models.

A crucial aspect that the guide seems to omit is the consideration of cultural particularities that transcend the western urban context of the country. The guideline lacks specific mention of how to address Schizophrenia in ethnic minority, indigenous and/or rural individuals. The literature has shown that, beyond the disorder and symptoms, there are significant differences in the quality of life of these groups, influenced by their belief systems, community practices and access to resources [25].

Otherwise, the Chilean Guidelines contrast to other countries treatment plans. For instance, the joint recommendations of the Canadian Psychiatric Association (CPA) and the Schizophrenia Society of Canada (SSC), which culminated in the publication of six clinical practice guidelines, address evaluation, diagnosis, pharmacotherapy, and the psychosocial approach in children, adolescents, and adults, as well as comorbidity with substance use and treatment for individuals with high clinical risk of psychosis [26].

Similarly, the OnTrackNY Program Treatment [27] is based on multidisciplinary units that collaborate closely with individuals diagnosed with a first psychotic episode. This program is grounded in essential pillars such as medication adherence, supported education and employment, family support and intervention, fostering illness self-management and recovery, social skills training, substance abuse treatment, coping skills training, behavioral activation, housing and income, trauma screening and treatment, and suicide prevention. These pillars are systematized in ten manuals that also address intercultural issues and culturally sensitive interventions in the first psychotic episode. These interventions have demonstrated their effectiveness in reducing the number of hospitalizations, increasing the likelihood of employment and/or return to formal education, and improving scores on general functioning scales [27, 28, 29, 30].

Analyzing the social context from cultural biology principles, it is relevant to reflect on whether this guide favors a matrix of trust and cooperation or if it reinforces a model of imposition based on hierarchical dynamics [16]. A person-centered care requires that practices and policies emerge from the acceptance and validation of the other as legitimate in their relational space [16, 17, 19].

### Ideologies and Effects of the Clinical Guidelines Discourse

The narrative regarding Schizophrenia in this guideline is given in terms of deficits and dysfunctions, emphasizing symptoms and risks, which could contribute to stigma in diagnosed people. In terms of decision-making power relations, the wording of the guide positions health professionals as the main actors in decision-making, relegating people with Schizophrenia and their families to a more passive role, although informed consent and the incorporation of the patient’s perspective are promoted. This discursive construction, while aligned with international standards, raises questions about its ability to encourage a truly participatory and empowering model of care that recognizes people with Schizophrenia as active agents in their recovery process.

The discourse of the guidelines directly impacts clinical practice and public perception of schizophrenia. By setting standards, it influences treatment decisions and promotes uniformity in the Chilean health system. However, the focus on drugs may restrict the use of broad psychosocial therapies that address the varied needs of patients.

On the other hand, the allusion to recommendations on social inclusion and family involvement in treatment may provide a positive counterbalance, although their effective implementation requires overcoming structural and cultural barriers. Ultimately, the guideline acts not only as a technical document, but also as a social actor that contributes to shaping attitudes toward Schizophrenia, an impact that can be both positive and limiting depending on the degree to which a holistic, person-centered approach is integrated.

From cultural biology, this biomedical emphasis can be interpreted as an expression of a control paradigm that seeks to solve problems from linear causality, ignoring the systemic nature of human beings [16].

### Tensions and Omissions in the Chilean Clinical Guide for Treatment from the First Episode Schizophrenia

The aim of this paper was to critically analyze the AUGE Clinical Guide for the Treatment of people from the first episode of Schizophrenia in Chile [13], based on the cultural biology of Maturana and Dávila framework [16, 17, 19], in order to identify the potentialities and challenges in the implementation of a person-centered approach. The analysis reveals that important challenges in the implementation of a truly person-centered approach still exist.

Analyzing the historical evolution of different versions of clinical guidelines, a shift from more structured and concise texts to a more comprehensive approach is observed [31, 32]. This latter approach emphasizes neurocognitive rehabilitation, social inclusion, and patient autonomy in decision-making as key elements in the treatment of Schizophrenia. Additionally, it broadens the focus on specific populations, such as those with treatment-resistant Schizophrenia and pregnant or breastfeeding people.

However, this modernization leads to the loss of important components in the formulation of the guidelines. There is a noticeable decline in evaluation mechanisms for the implementation of these guidelines in health services, a reduced emphasis on primary prevention and early detection of the disorder, and a decrease in the extent and depth of psychosocial strategies for managing Schizophrenia across its different phases. Specifically, there’s a transition from detailed planning of individual and family interventions to merely listing evidence-based therapy types. While this change may grant greater autonomy and flexibility to experienced healthcare providers, it could create difficulties and complications in interventions carried out in less complex health centers.

As a result, pharmacological interventions are most consistently implemented at the expense of the other areas of intervention equally necessary for the personal recovery and quality of life of people with Schizophrenia. Thus, there is constant tension between the predominant biomedical model, which emphasizes the use of pharmacotherapy, and the need to integrate social, cultural and human rights dimensions in mental health care, as promoted by international organisms such as the World Health Organization [33]. This suggests the need for a deeper transformation in the training of health professionals and in the institutional culture to achieve care that empowers people with Schizophrenia and promotes their autonomy in the recovery process.

Regarding the social context, it emerges that the Chilean Guidelines present notable differences in comparison with consolidated international models. It is crucial to analyze these discrepancies in light of the recommendations of the CPA and the SSC [26], as well as the OnTrackNY treatment program [27], which offer detailed clinical guidelines for the assessment, diagnosis, pharmacotherapy and psychosocial approach in diverse populations, including those with comorbidity for substance use and comorbidity and high risk for psychosis, and are based on multidisciplinary units and comprehensive treatment pillars that have demonstrated efficacy in reducing hospitalizations and improving overall functioning [28, 29, 30].

Another relevant omission in the guide is the consideration of cultural, ethnic and rural context differences, given that the literature supports that factors such as belief systems, community practices and access to resources significantly influence the quality of life and recovery of these individuals. A specific section should be included in a future guide, covering culturally adapted strategies, integrating approaches from intercultural health and experiences of models of care in indigenous and rural communities.

The disparity between the Chilean Guidelines and these international models raises questions about the need to incorporate or adapt proven strategies to optimize the care of patients with schizophrenia from the first episode in the Chilean context. Discussion of these differences may guide future research and the revision of existing guidelines to ensure more comprehensive and effective care.

It is essential that future Clinical Guidelines adopt a more holistic approach that recognizes the complexity of Schizophrenia and values the individual experience of each patient, as well as a greater development and elaboration of the sections on non-pharmacological interventions for the disease, considering that only 5 pages of the document correspond to psychosocial interventions. In this regard, there are promising developments related to psychosocial interventions for Schizophrenia. These include mindfulness, training of social and cognitive skills, acceptance and commitment therapy, individual and group peer support, and compassion-focused therapy [34].

### Recommendations on the Formulation of Clinical Guidelines From the Cultural Biology Framework

The analyzed clinical guidelines represent an important progress in the care of mental health services provided in Chile. However, they remain grounded in a paradigm that fails to position emotional, subjective and interpersonal relationships as fundamental components of the recovery process. From the Cultural Biology perspective, several key points are proposed for the formulation of the guide, in order to approach Schizophrenia as a relational condition beyond the traditional biomedical understanding.

Regarding screening and diagnostic confrontation, the application of standardized clinical criteria needs to be complemented by components that recognize the diagnosis as a consensual coordination of actions and meanings between the professional, the patient and his or her affective network. Thus, the diagnosis should arise from a dialogic process that validates the subjective experience of the user and contextualizes the symptoms in their life history and environment.

On the other hand, from Cultural Biology love is understood as the emotion that confirms that everyone is a legitimate other in coexistence [16], so the reconstruction of bonding networks based on mutual legitimacy is central to recovery. In this sense, the creation and strengthening of an affective companion within mental health teams is recommended, whose main function is emotional containment, subjective recognition and the patient’s relational sustainability. This figure can be performed by professionals or trained members of the community.

Regarding the Psychoeducation process, from Cultural Biology it is understood that mental health implies reintegrating into meaningful conversational spaces [16, 17, 18, 19, 20, 21], so it is suggested that Psychoeducation on Schizophrenia to patients and family members should include dialogic spaces where they lived experience is legitimized, and a shared meaning is built from active listening, emotional validation and personal narratives.

Another key point is the incorporation of open and communal physical spaces in mental health facilities, to encourage expression, encounter and symbolic construction through art, writing and music, in order to create a common world environment, where the subject returns to inhabit a shared reality. From Cultural Biology, psychic suffering can be understood as a rupture of the shared world. Restoring symbolic and affective communication is key to the reintegration of people with Schizophrenia [16, 17, 19, 21].

Additionally, include training for all professionals in the principles of observation as a relational act, language as a constructor of realities and emotions as the basis of knowing. This training, which should be transversal and continuous, can contribute to transforming the clinic from its foundations.

Finally, it is crucially important to include people with the lived experience of Schizophrenia in the committees that review and update the Guidelines, providing a perspective from the subjective and social experience of the disorder. The construction of clinical knowledge should integrate the diversity of observers involved in the experience. This is the only way to validate the legitimacy of the other as a subject of knowledge [16].

## Conclusion

The aim of this paper was to critically analyze the Chilean Clinical Guide for the Treatment of Schizophrenia in order to identify the potentialities and challenges in its principles. The analysis reveals that exists important challenges in the implementation of a truly person-centered approach.

Cultural biology offers a valuable perspective for rethinking public mental health policies in Chile and Latin America. Its emphasis on the co-construction of reality, language as the central axis of social interaction and love as the engine of human coexistence, provides tools to build a more just, equitable and humane health system. It is crucial to generate spaces for dialogue and critical reflection that allow transforming care practices and building a more human, fair and inclusive health system. Similarly, an interdisciplinary dialogue that integrates knowledge from different areas of knowledge is necessary to generate public policies that promote social inclusion, citizen participation and respect for the human rights of people with Schizophrenia. This is the only way to move towards a more just and equitable society that values diversity and promotes the well-being of all people.
